# Health Tract, No. 214

**Published:** 1864-09

**Authors:** 


### POTATOES.

Some soils produce several hundred bushels of the white commonly called the Irish potato, and there is such a general taste for them that they are likely to continue to be a very common article of diet, although not as valuable or any more healthful than many other qualities of food. Three fourths of the potato is water, so that of one hundred pounds only twenty-five pounds are nutriment; the rest is waste. Almost the entire nutriment is contained within a quarter of an inch of the surface, immediately under the skin; hence by peeling, the very best part of it, and nearly all that is valuable, is wasted. Only the outer skin should be removed, that which is disposed to peel off after boiling.

It is said that potatoes grown in France are entirely free from disease by having been planted in June instead of April, so as to escape the alternations of heat and cold by the changeable weather of spring.

The tendency of potatoes to sprout in the early spring is reported to be prevented in Scotland, and by so doing, their full edible qualities are preserved, and "mealy" potatoes can be had all summer from the previous year's growth. The experiment costs but little, and is worthy of being tested by every one who doubts its efficacy. Obtain from a druggist one ounce of liquor of Ammonia, (hartshorn) to a pint of water; let the potatoes be immersed in this mixture four or five days; dry them. Their substance is thus consolidated, and much of their moisture extracted without the slightest injury for all table qualities, but their vegetative power is forever destroyed. If spread out after immersion, so as to be well dried, they will keep good for ten months.

Baked potatoes are easily digested, requiring only two hours and a half, but one hour longer if boiled. If baked in the ashes and eaten with butter and salt, they are sweeter and more healthful than by any other mode of preparation. The sprouts of potatoes uncovered with earth contain solanum, a powerful poison, the potato becoming green, and are then unfit for even animals. To have mealy potatoes for the table, boil them until the fork easily penetrates; pour off all the water; cover the vessel with a cloth near the fire, until "steamed" dry.

